# Parents are a Drag: Long-Lived Birds Share the Cost of Increased Foraging Effort with Their Offspring, but Males Pass on More of the Costs than Females

**DOI:** 10.1371/journal.pone.0054594

**Published:** 2013-01-30

**Authors:** Shoshanah R. Jacobs, Kyle Hamish Elliott, Anthony J. Gaston

**Affiliations:** 1 Department of Integrated Biology, Guelph, Guelph, Ontario, Canada; 2 Department of Zoology, University of Manitoba, Winnipeg, Manitoba; 3 Environment Canada, National Wildlife Research Centre, Ottawa, Ontario, Canada; University of Manchester, United Kingdom

## Abstract

Life history theory predicts that parents will balance benefits from investment in current offspring against benefits from future reproductive investments. Long-lived organisms are therefore less likely to increase parental effort when environmental conditions deteriorate. To investigate the effect of decreased foraging capacity on parental behaviour of long-lived monogamous seabirds, we experimentally increased energy costs for chick-rearing thick-billed murres (*Uria lomvia*). Handicapped birds had lighter chicks and lower provisioning rates, supporting the prediction that long-lived animals would pass some of the costs of impaired foraging ability on to their offspring. Nonetheless, handicapped birds spent less time underwater, had longer inter-dive surface intervals, had lower body mass, showed lower resighting probabilities in subsequent years and consumed fewer risky prey items. Corticosterone levels were similar between control and handicapped birds. Apparently, adults shared some of the costs of impaired foraging, but those costs were not measurable in all metrics. Handicapped males had higher plasma neutral lipid concentrations (higher energy mobilisation) and their chicks exhibited lower growth rates than handicapped females, suggesting different sex-specific investment strategies. Unlike other studies of auks, partners did not compensate for handicapping, despite good foraging conditions for unhandicapped birds. In conclusion, parental murres and their offspring shared the costs of experimentally increased foraging constraints, with females investing more than males.

## Introduction

Life-history theory predicts that iteroparous organisms will balance behaviours that lead to improvement in current reproductive success against behaviours that will lead to improvements in subsequent reproduction [1], [2], [3]. Long-lived iteroparous species likely safeguard self-maintenance because a small reduction in adult survival can greatly reduce lifetime fitness [4], [5]. In contrast, short-lived species that are less likely to survive until another reproductive event are more likely to sacrifice survival and condition to increase current reproductive output [6], [7].

Long-lived adult seabirds may buffer small changes in environmental conditions by altering their behaviour to maintain offspring provisioning rates [8], [9]. When foraging costs increase beyond a threshold, adult seabirds pass an increasing proportion of the additional costs along to their chick to maintain their own condition [10], [11], [12]. Where this threshold lies varies among species, environments and adult physiological conditions [13], [14], [15]. In some cases, adult seabirds have a fixed level of investment [16], [17], [18], [19] while others have flexible investment adjusted according to offspring demand, sometimes leading to reduced adult survival [20], [21], [15].

Adult seabirds use several cues to regulate their breeding behaviour. Stress hormones (e.g. corticosterone) are elevated in response to food shortage, and elevated levels trigger begging in chicks and self-maintenance behaviour in adults [22], [23], [24]. Similarly, the level of endogenous energy stores (lipids) may govern parental investment decisions, with individuals unwilling to increase effort in raising young when their stores drop below a critical threshold [25], [20], [26]. Declines in endogenous stores can trigger changes in behaviour (e.g. abandonment, reduced energy delivery rates) through changes in hormone levels (e.g. corticosterone or prolactin, [23], [24], [27].

While specific nutrients may play a role in chick development, chick growth is primarily determined by energy intake [16]. Thus, where predation on adult birds is low, parental investment can be measured in terms of the transfer of energy stores (lipids) to the offspring via adult energy expenditure to provision offspring [28]. As plasma neutral lipids are the main form of lipid mobilisation in birds (as opposed to structural lipids, such as phospholipids [29], [30], [27], an index of investment can be obtained by sampling plasma neutral lipids. High levels of circulating neutral lipids are associated with high energy expenditure (chick-rearing murres [26]; small fish [31]; birds [27], [32], [33]. Plasma neutral lipid level is associated with parental mass loss during chick-rearing [34], as lipids are mobilised to fuel increased energy spent flying, and parental mass during chick-rearing is negatively correlated with the mass gain of chicks [35], [36]. Plasma lipids are potentially a better measure of energy mobilisation than body mass because changes in body mass can represent changes in non-lipid portions [37], [26].

Investment levels can also vary between the sexes within a species, as the partner with the lowest initial investment in gametes (males) has the lowest subsequent investment (Bateman`s principle, *sensu* Bateman 1948). For long-lived socially monogamous species with biparental care, sex-stereotyped differences in parental investment often maximise both partners' lifetime reproductive success (e.g. risk-partitioning [38]). Having each partner specialise in a different tactic may increase both partners' fitness. Similarly, male seabirds often follow behavioural rules consistent with a fixed investment strategy and females often follow rules consistent with a flexible investment strategy [39], [40], [38]. Thick-billed murres (*Uria lomvia*) are an interesting species to examine for sex-specific investment strategies because, unlike most animals, males make a larger investment than females after the chick departs from the colony, accompanying the chicks for a month-long period of male-only care [41], [38]. Male-only chick care after departure is believed to affect provisioning behaviour at the colony, as males forage at different times of day, at different locations and on different prey than females, presumably because the male specialises in risk-averse prey items that do not require long-distance flights and that the male can continue to capture while it cares for the flightless chick at sea [42], [10], [41], [28].

Understanding how animals react to increased energy expenditure is an essential part of understanding how they will react to changes in environmental conditions. Due to earlier ice break-up in Hudson Bay, seabirds in northern Hudson Bay have switched from provisioning their chicks with large, ice-associated arctic cod (*Boreogadus saida*) to smaller capelin (*Mallotus villosus*) that are characteristic of sub-Arctic waters of the North Atlantic [43]. In consequence, chicks grow less quickly [44], [45]. Flight costs dominate energy budgets for auks, and although birds fly farther for larger prey, the energy costs for delivery of many small items is still greater than a single large item [46]. Thus, increased energy expenditure is expected to be one consequence of environmental change at our arctic study site. Furthermore, as judged by adult and chick mass, conditions at our study site (including our study years) were consistently among the best of any murre colony in the Canadian Arctic [47], [35], [36], [48]. Therefore, murres at our study site are likely to show flexibility in self-investment and chick-provisioning rates in response to reduced feeding rates, whereas murres at study sites with already marginal feeding rates might simply abandon.

Past studies of flexibility in parental investment in seabirds have focused on species that maintain high levels of endogenous stores (e.g. petrels [17], [18] or have large clutch sizes (e.g. gulls [3]). We studied flexibility in parental strategies of thick-billed murres, a species with a single-egg clutch. Due to small size and high wing loading, flight costs are high and endogenous stores are low relative to daily energy expenditure [49], [26], and murres are likely to be relatively inflexible in investment [11], [12]. We experimentally increased drag, buoyancy or wing-loading for murres rearing nestlings to simulate some aspects of unfavourable environmental conditions; increased drag reduces time available for foraging during each dive and reduces energy intake per energy expended [50], [51]. While adding mass may only alter flight costs and clipping wings may increase flight but decrease dive costs (assuming wing size is a compromise between optimal size for flight and diving [52]), adding drag would likely increase both flight and dive costs. We predicted that handicapped males would maintain their own condition (body mass, neutral lipids, corticosterone levels) while allocating fewer resources to their chicks (indicated by reduced chick growth rates and adult lipid delivery rates), with the potential for compensation by the partner (fixed investment [10]), whereas handicapped females would tend to sacrifice their own condition to increase the well-being of their chicks (flexible investment).

## Methods

### Ethics Statement

The protocols described here were approved by the Animal Care Committee of the University of Ottawa, Canada, under the permit BL-172.

All manipulations and observations were conducted during the chick rearing periods of 2003 (27 July to 12 August: corticosterone, lipid and adult/chick body mass studies; single handicap and wing-clipping treatments), 2004 (31 July to 13 August: adult/chick body mass, feeding watch and dive behaviour studies; single and double handicap treatments) and 2005 (6 to 9 August: feeding watch and dive behaviour studies; single handicap treatment only) at the Z study plot on Coats Island, Nunavut in the Canadian Eastern Low Arctic [43], [44]. We monitored the plot daily to determine chick hatch dates. Chicks averaged 4.5±0.4 d at the start of all manipulations, which lasted for two weeks. All birds were sexed genetically using PCR after the field season (see details in [38]). In August 2006, we attempted to resight all birds used during handicap experiments. One member of each pair was handicapped, so for each experiment we had three experimental groups: handicapped birds, partners of handicapped birds and control birds (neither partner handicapped). All handicaps were removed after 14 days.

### Handicaps

We experimentally increased energy expenditure by increasing both drag and buoyancy (floater experiment) and wing-loading (wing-clipping) and a summary of manipulations is presented in Table 1. Drag is more important than buoyancy for determining underwater costs in murres, and impacts diving more than flying [50], [53]. Increasing energy costs reduce dive depth and duration while increasing surface pauses because oxygen is used up quicker, allowing feeding birds less time to access prey [50], [54], [37]. Thus, we designed the floaters to primarily challenge dive efficiency and the wing-clipping to primarily challenge flight efficiency.

**Table 1:** Summary of methods used in the current paper.

Handicap method	Year	Measure	Results
Single floater	2003–04	Chick growth rates	Decreased
Single floater	2004	Chick-provisioning rates	Decreased
Single floater	2004	Partner’s chick-provisioning rates	No change
Single floater	2003–04	*Adult body mass*	Decreased
Single floater	2003	*Adult plasma lipids*	No change, except increased for males on day 6
Single floater	2003	*Adult corticosterone levels*	No change
Single floater	2004–05	Dive depth and duration	Decreased
Single floater	2003–07	*Resighting rate*	No change
Wing clipping	2003	Chick growth rates	Decreased
Wing clipping	2003	*Adult body mass*	No change
Wing clipping	2003	*Adult plasma lipids*	No change
Two floaters	2004	Chick growth rates	Decreased
Two floaters	2004	*Adult body mass*	Decreased
Two floaters	2004–07	*Resighting rate*	Decreased
Two floaters	2004	Partner’s chick-provisioning rates	No change

Values are considered to have changed only if results were statistically significant. Measures that primarily impact the adult are shown in italics.

Floater handicaps were hollow plastic fishing floaters (mass = 1.0 g) with the seams sealed by epoxy to ensure that they did not fill with water. From a month prior to the experiments, observations were made daily to determine the date of hatching at each breeding site [55], [56]. On 27 July 2003, 16 adults were caught for the experimental group (10 females and 6 males) and 15 individuals were caught for the control group (7 females and 8 males). We attached a single floater to experimental birds in 2003. On 29 July 2003, we also handicapped 7 males and 9 females (with 7 males and 9 females as controls) by shortening their primary feathers on both wings by 20 mm. As these wing-clippings had smaller effects on adults than the fishing floaters (see Results), we did not repeat the wing-clipping experiments in 2004. On 31 July 2004, 15 adults were caught for the two handicap treatment (floater on both legs; 7 females and 8 males), 15 individuals for the one handicap treatment (floater on one leg; 6 females and 9 males), and 14 individuals as controls (no handicap; 7 females and 7 males). On 6 August 2005, 8 adults were caught for the one handicap treatment (handicap on one leg; 6 males and 2 females). In all years, the treatments were applied randomly and sex determined after the field season using PCR, as described elsewhere [38].

In 2003 and 2004 we caught the chicks at each experimental breeding site by hand on the first day of the experiment and on days 6 and 12 thereafter, weighed them with a Pesola™ scale to the nearest 1 g, measured their wing chord to the nearest 1 mm and replaced them at their site within 3 min of capture [55], [56]. On day 0, we banded the chicks with a metal band and in 2004, added a small dot of coloured nail polish to their culmen for easier identification during feeding watches. The day after all chicks were measured, we captured and weighed adults, obtained a blood sample within 3 min of capture (in 2004 only a few µL for sex identification) and released them within 5 min of capture [57]. In 2003, approximately 3 mL of blood were taken using a syringe and 23 G butterfly needle. The blood was transferred immediately to a 3 mL heparinized vacutainer, centrifuged within 5 h of collection, frozen in a propane freezer (−20°C) and transported in a dry nitrogen shipper at the end of the season for laboratory analyses.

### Diving Experiment

In 2004 (N = 2 male and 3 female controls; 3 male and 4 female handicapped birds) and 2005 (N = 5 male and 2 females for both controls and handicapped birds), we attached LOTEK 1100LTD time-depth recorders (TDRs; Lotek Marine Technology, St. John’s, Newfoundland, Canada; mass = 4.5 g; diameter = 1 cm; equivalent to 0.9% of murre cross-sectional area and 0.45% of body mass; accuracy = ±2 m; see [58] for details) to the leg bands of randomly-selected birds with and without single floater handicaps. The TDRs were close to neutrally buoyant and had a cross-sectional area about 17% that of the floaters. Furthermore, the TDRs were attached snugly to the leg while the floaters trailed behind the bird, so the floaters were more likely to create flow disruptions that increased drag. The TDRs were attached to individuals breeding at a different area of the colony than the other experiments. After approximately 24 h, the birds were recaptured and all gauges and floaters removed. Whereas back-mounted TDRs reduce provisioning rate, dive depth and time, and mass gain, our leg-mounted TDRs had no significant effect on any of these parameters (see data presented in [10], [50], [54]). As is standard in studies of diving, we restricted analyses to 04∶00–21∶00 to avoid times of day when dive depths were reduced due to darkness and because chicks are rarely fed at those times [58], [46]. At-night, dive depth and duration within an individual decreases by an order of magnitude, and were we not to exclude those times, our values for average dive depth and duration for a particular individual would merely reflect the amount of time spent diving during darkness. Outside of those times, there is no difference in dive duration or depth between males and females [38]. All studies of dive behaviour occurred on birds with chicks 3–15 d old as feeding rates do not vary during that age range [58], [38].

### Feeding Watches

During the 2004–05 experiment, feeding watches were conducted on handicapped individuals during times when data from previous years showed that most feeding occurred (validated in [59]. On days 2–5 and 8–10 post-handicapping (2004) and days 2–3 post-handicapping (2005), feeding watches were conducted in the early morning (4∶30–9∶30) and in the evening (16∶00–20∶00). We marked one partner of each pair with a marker so that we could recognize individuals; colour band combinations were also used for identification of individuals. From a blind <5 m from the subjects, we recorded all feeds of experimental birds and their partners, including the time of delivery, the species delivered, and the approximate length (measured by comparison with the length of the culmen). We minimized bias in estimated food delivered following the suggestions of [59] to add 20 mm to the estimated length of each fish and to exclude feeding watch observations during the nighttime, when misidentifications increase. In addition, all deliveries made by the partner of the individual used in the experiment were recorded. From the estimated fish length, we determined energy and lipid content using species-specific mass-length, energy content and lipid content relationships for our study site [51], Table 2). We restricted analyses to chicks aged 3–15 d because energy content delivered to offspring does not vary with age over that age range [58]. Birds foraging on prey items that are variable in space and time were considered to be using a risk-prone strategy [60]. In our study, we considered birds feeding on invertebrates and small capelin to be risk-averse, as encounter rates for these prey (“less-risky”) are relatively constant across space and time [14]. Schooling fish (large capelin, sand lance and Arctic cod) were considered risk-prone prey items (“more-risky”), as encounter rates for those are highly variable (see [61], [38] for more details).

**Table 2:** Lipid content (g) of Arctic prey species, as determined using the methods of Jacobs et al.

Species	A	b	R^2^	Lipids per mass
Arctic cod (*Boreogadus saida*)	0.107	0.701	0.80	0.047±0.010
Capelin (*Mallotus villosus*)	0.377	1.271	0.74	0.062±0.028
Sculpin (*Triglops* sp.)	0.057	0.988	0.81	0.055±0.024
Sandlance (*Ammodytes* sp.)	0.067	0.965	0.85	0.063±0.004
Fish doctor (*Gymnelus* sp.)	0.086	0.000	0.85	0.021±0.056
Blennies (*Leptoclinus maculatus, Eumesogrammus praecisus, Stichaeus punctatus*)	0.930	−0.579	0.71	0.022±0.011
Shrimp (Decapoda sp.)	0.131	0.000	0.85	0.027±0.004
Squid (*Gonatus fabricii*)	0.079	0.000	0.85	0.015±0.002
Amphipods (*Parathemisto libellula*)	0.044	0.000	0.82	

(2009). Lipid content, *L*, is related to body mass, *M*, by the formula *L* = a*M* ^b^. Also shown are lipids (in g) per gram of total body mass (± SE). Fish classification follows Elliott and Gaston (2009).

### Lipid and Corticosterone Analyses

Lipid analyses followed established protocols and standards [57], [34]. Briefly, plasma was added to Folch reagent (2∶1 v/v) and filtered [57]. Neutral lipids, non-esterified fatty acids and phospholipids were separated, re-suspended in chloroform and transferred into columns. The neutral lipid fraction was eluted by flowing chloroform:isopropanol (2∶1 v/v) through the columns. Fats were re-suspended in an acetyl chloride solution (7.2 mL acetyl chloride in 100 mL methanol) and incubated at 90°C for 2 h, and then resuspended in methanol. In each case, the solvents were evaporated under N_2_ at 70°C. The fats were resuspended in isooctane and transferred to gas chromatograph autosampler tubes. All samples were then analyzed using gas chromotography (Hewlett-Packard 5890 series II with Hewlett-Packard 7673 autosampler and flame-ionization detector). The retention times of the fatty acids were compared with those from known standards.

To measure corticosterone concentrations, serum samples were extracted using dichloromethane and run on a single radioimmunoassay following established protocols [24], [62]. Intra-assay variability was 14%.

### Statistical Analyses

We used general linear models to analyse dive behaviour, adult masses, energy and lipid delivery rates, plasma neutral and total lipid concentrations, corticosterone and chick growth rates, after controlling for individual. Dive behaviour was calculated for a continuous 18 hr period of diving (excluding night as described earlier) for both control and handicapped birds. For dive behaviour and feeding rates, we used general linear mixed models to account for individual variation. To account for chick age and the nonlinear growth of the chicks, we used a cubic spline to estimate chick mass in each year from all data combined. To estimate relative chick growth rates, we used individual chick mass at a particular age minus expected chick mass at a particular age derived from the spline. Values for all other parameters were calculated relative to initial values (average chick age = 4.5 d ±0.4 d), with measurements occurring at day 6 (chick age = 10.5 d) and 12 (chick age = 16.5 d). Thus, statistical analyses were made only for those measurements at day 6 and 12, expressed relative to measurements for the same individual at day 0. We included chick age as a covariate, although variation in chick age at each sampling date was small. We considered interactions between sex, year and treatment (but not all three together), and considered only the full models. If the general linear model provided a significant result (P<0.05), then we used post-hoc t-tests to compare among the different groups. We followed the recommendations of [63] and did not simplify models using stepwise regression. Rather, we considered only the full model with all interactions [63]. To combine resighting probabilities for 2003 and 2004, we corrected resightings in 2004 by raising proportions to the power of 0.67 to account for higher probability of resighting birds from 2004 than from 2003 in 2006 (2 = winters 2004–06; 3 = winters 2003–06, so corrected by 2/3). We used Fisher’s exact test to compare the number of birds resighted to the number of birds not resighted for handicapped relative to control birds. We tested for normality (Shapiro-Wilks test) and homogeneity of variance (Levine’s test) prior to using parametric statistics. All statistical analyses were conducted using R 2.4.1.

## Results

### Adult Mass, Adult Resighting

**Figure 1:**
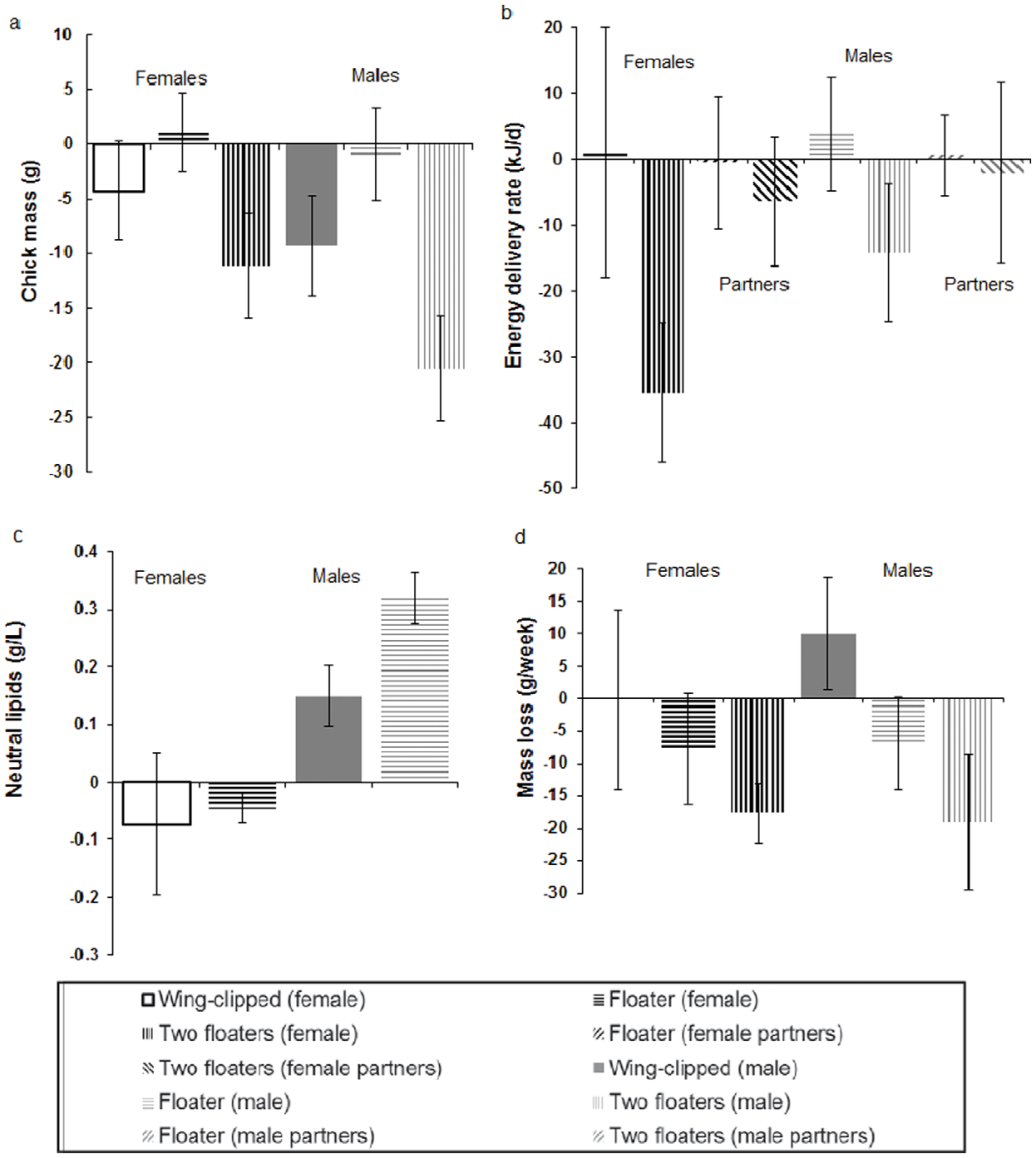
The effects of floaters and wing clipping of breeding Thick-billed Murres. **a.** Residual on chick age of chick mass, relative to residuals at the start of the experiment; **b.** Energy delivery rates, **c.** Plasma neutral lipids at six days and **d.** mass loss per week of parental thick-billed murres at Coats Island 2003–2005. All values are shown relative to control birds (average for experimental birds across all individuals – average for control birds across all individuals with the same sex) and SE bars include total SE_T_ propagated from control SE_C_ and experimental SE_E_ using SE_T_^2^ = SE_C_^2^+ SE_E_^2^. Statistical results are shown in Table 3. Females shown in black and white (left side of each graph); males shown in grey and white (right side of each graph).

There was no initial difference in body mass among treatments (2003: *F*_2,45_ = 0.8, P = 0.45; 2004: *F*_2,40_ = 1.28, P = 0.29), between sexes (t_74_ = 1.67, P = 0.10) or between years (t_74_ = 1.03, P = 0.31). As handicapped birds lost more mass than control birds by day 12, handicapped birds weighed less than control birds on day 12 (Table 3, Fig. 1). To account for inter-individual differences in mass trajectories, we compared body mass within the same individuals at days 6 and 12 relative to the start of the experiment. There was no difference in body mass change between day 6 and day 12 (t_84_ = 1.52, P = 0.13), so we averaged values across these two days. Body mass change differed between the three treatments after accounting for sex (Table 3, Fig. 1d). A post-hoc t-test showed that after accounting for sex there was a significant difference in body mass loss for birds that were doubly-handicapped but not for singly-handicapped birds (Table 3, Fig. 1d).

**Table 3:** Values of (± SE) and statistical variation (GLM) in response variables of parental thick-billed murres at Coats Island relative to the independent variables of year, sex and handicap.

	df	First year^1^	Second year^1^	F(P)	Male	Female	F(P)	Wing-clipped	Two floaters	Floater	Control	F(P)	F^5^(P)	F^6^(P)	F^7^(P)	F^8^(P)	R^2^
Dive depth (m)	1,17	46.2±7.2	53.6±3.2	1.11 (0.31)	48.2±8.2	54.5±9.0	0.34 (0.57)			**42.1±5.7**	**73.2±6.2**	**10.9 (0.004)**	1.78 (0.2)	0.62 (0.44)	0.73 (0.40)	0.07 (0.79)	0.79 (0.30)
Dive duration (s)	1,17	42.1±3.4	44.2±4.6	1.71 (0.21)	38.6±3.8	44.6±4.0	1.32 (0.27)			**37.2±1.6**	**62.2±2.1**	**9.21 (0.008)**	2.34 (0.14)	0.55 (0.47)	0.64 (0.43)	0.05 (0.83)	0.80 (0.30)
Surface pause (s)	1,17	0.5±5.2	−3.2±8.6	1.04 (0.32)	1.2±2.1	−2.0±3.7	0.22 (0.65)			−**10.4±6.0**	**12.2±5.2**	**18.1 (0.001)**	3.21 (0.09)	1.21 (0.29)	1.58 (0.23)	0.26 (0.62)	0.84 (0.26)
Time underwater (min)	1,17	194±16	201±13	0.77 (0.39)	196±14	207±10	0.84 (0.37)			**171±19**	**221±11**	**14.5 (0.001)**	1.99 (0.18)	0.75 (0.40)	0.89 (0.36)	0.08 (0.78)	0.82 (0.27)
Delivery rate (kJ/d)	1,53				162±12	195±18	1.83 (0.18)		**129±21**	**198±26**	**195±19**	**13.8 (<0.001)**	3.65 (0.06)				0.81 (0.29)
P:Delivery rate (kJ/d)	1,53				163±15	208±17	2.78 (0.10)		202**±**18	195**±**13	195±19	4.01 (0.05)	1.56 (0.22)				0.22 (0.69)
Lipid delivery rate (g/d)	1,53				2.38±0.18	2.82±0.29	1.39 (0.24)		**1.80±0.28**	**2.85±0.31**	**3.14±0.64**	**9.71 (0.003)**	**4.25 (0.04)**				0.76 (0.33)
P:Lipid delivery rate (g/d)	1,53				2.99±0.25	3.25±0.25	1.22 (0.27)		3.56±0.18	3.10±0.22	3.14±0.64	3.37 (0.07)	1.33 (0.25)				0.16 (0.74)
Chick growth (g)	1,63	−0.65±3.48	2.19±2.37	0.57 (0.45)				−**2.87±6.41**	−**3.38±3.32**	−**2.08±3.25**	**7.74±3.74**	**13.1 (<0.001)**			**6.93 (0.01)**		0.85 (0.26)
Mass loss (g/week)^2^	1,45	−19.1±6.2	−19.6±14.9	0.30 (0.59)	−17.8±6.1	−20.4±10.4	0.19 (0.67)	−14.4±13.5	−28.3±7.1	−23.1±6.0	−16.2±5.7	0.60 (0.44)	1.01 (0.32)	0.42 (0.52)	0.69 (0.41)	0.05 (0.82)	0.08 (0.82)
Mass loss (g/week) 3	1,32	−37.1±5.5	−37.9±6.3	0.24 (0.63)	−**20.4±10.4**	−**47.4±7.4**	**100.8 (<0.001)**	−16.0±20.1	−**52.1±6.2**	−**45.8±11.7**	−**33.0±7.5**	**20.5 (<0.001)**	1.04 (0.32)	0.33 (0.57)	0.38 (0.54)	0.91 (0.35)	0.88 (0.22)
CORT (ng/mL)^2^	1,13				−0.12±0.21	−0.18±0.12	1.03 (0.33)			−0.18±0.14	−0.13±0.15	0.90 (0.36)	0.20 (0.66)				0.07 (0.83)
CORT (ng/mL)^3^	1,13				−0.82±0.22	−0.38±0.23	2.5 (0.14)			−0.62±0.17	−0.63±0.17	0.24 (0.63)	0.49 (0.50)				0.06 (0.44)
Neutral lipid (g/L)^2^	1,23				0.22±0.13	0.11±0.04	0.01 (0.92)	0.24±0.04		0.16±0.09	0.16±0.14	0.76 (0.39)	**6.87 (0.02)**				0.54 (0.48)
Neutral lipid (g/L)^3^	1,8				−0.12±0.12	0.51±0.75	0.97 (0.35)	−0.22±0.20		−0.32±0.23	0.24±0.22	0.81 (0.39)	1.72 (0.23)				0.12 (0.77)
Total lipid (g/L)^2^	1,23				−0.41±0.63	−0.03±0.58	0.01 (0.92)	−1.18±0.61		−0.74±0.59	.0.32±0.59	0.06 (0.81)	1.70 (0.21)				0.09 (0.81)
Total lipid (g/L)^3^	1,6				−1.22±0.59	0.84±0.95	0.81 (0.40)	−2.17±4.17		−1.29±1.47	−0.31±0.58	1.10 (0.33)	1.61 (0.25)				0.11 (0.79)
Resighting (+3 years)		56%	65%	(0.31)	60%	60%	(0.22) 4		**40%**	**55%**	**65%**	**(0.04)** 4					

P = partners of handicapped birds. Note that adult blood sample analyses were not obtained in 2004 and adult delivery rates were not obtained in 2003. Statistically significant relationships are shown in bold (post-hoc t-test). Surface pauses and chick growth are residuals on dive duration and chick age, respectively.

1 The first year of experimentation was 2003 and the second year of experimentation was 2004 for all experiments except the dive experiments. The first year of experimentation was 2004 and the second year of experimentation was 2005 for the dive experiments.

2 Day 6 after attachment of handicaps.

3 Day 12 after attachment of handicaps.

4 P-values from Fisher’s exact tests.

5 Interaction between sex and experiment.

6 Interaction between sex and year.

7 Interaction between year and experiment.

8 Three-way interaction.

Resighting probabilities in 2003 (56%) and 2004 (65%) did not differ significantly (P = 0.31 from Fisher’s exact test), nor did males and females differ (both 60%). Doubly-handicapped birds had a lower subsequent resighting probability than controls (Table 3) but singly-handicapped birds did not differ significantly from controls (Table 3).

### Plasma Lipids and Corticosterone

At the start of the experiment plasma and neutral lipid levels and corticosterone did not vary between control and experimental birds or between males and females (Table 4). To account for inter-individual differences, we compared plasma lipid levels within the same individuals at days 6 and 12 relative to the start of the experiment. There was no difference in the change in total plasma lipid levels between handicapped (single floater) and control birds at day 6 (Table 3) or day 12 (Table 3). There was no difference in the change in plasma neutral lipid levels between handicapped (single floater) and control birds at day 12 (Table 3). However, there was a significant interaction between sex and treatment at day 6 (Table 3, Fig. 1c) with neutral lipids higher in handicapped males than in either handicapped females or control males (interaction term). There was no difference in total lipids (Fig. 1).

**Table 4:** Total plasma and neutral lipid concentration (± SE) of adult male and females sampled on day 0 at the start of the experiment.

	Control	Experimental	df	t	P
Neutral Lipid (g/L)	0.41±0.03	0.39±0.05	26	0.35	0.73
Total lipid(g/L)	1.37±0.12	1.36±0.10	30	0.08	0.93
Corticosterone (mg/mL)	13.3±1.8	16.4±1.5	30	1.34	0.19
	**Males**	**Females**			
Neutral Lipid(g/L)	0.43±0.04	0.35±0.04	26	1.43	0.16
Total lipid (g/L)	1.34±0.12	1.39±0.08	30	0.30	0.77
Corticosterone (mg/mL)	17.1±2.1	13.0±1.7	30	1.81	0.08

On day 0 corticosterone levels did not differ between experimental and control birds or between males and females (Table 4). Corticosterone levels were higher early in chick-rearing than later (average decline between day 0 and day 12 = 4.2±1.8 ng/mL, paired t_26_ = −2.31, P<0.02). To account for the inter-individual differences in those trajectories, we compared corticosterone levels within the same individuals at days 6 and 12 relative to the start of the experiment. There was no difference in the decline in corticosterone levels between handicapped (single floater) and control birds at day 6 or day 12 (Table 3).

Wing-clipping did not affect adult body mass or neutral lipid measures to the same degree as adding floaters, although effect sizes were similar for chick growth rates (Table 3, Fig. 1).

### Diving Experiment, Feeding Rates and Chick Growth Rates

**Figure 2:**
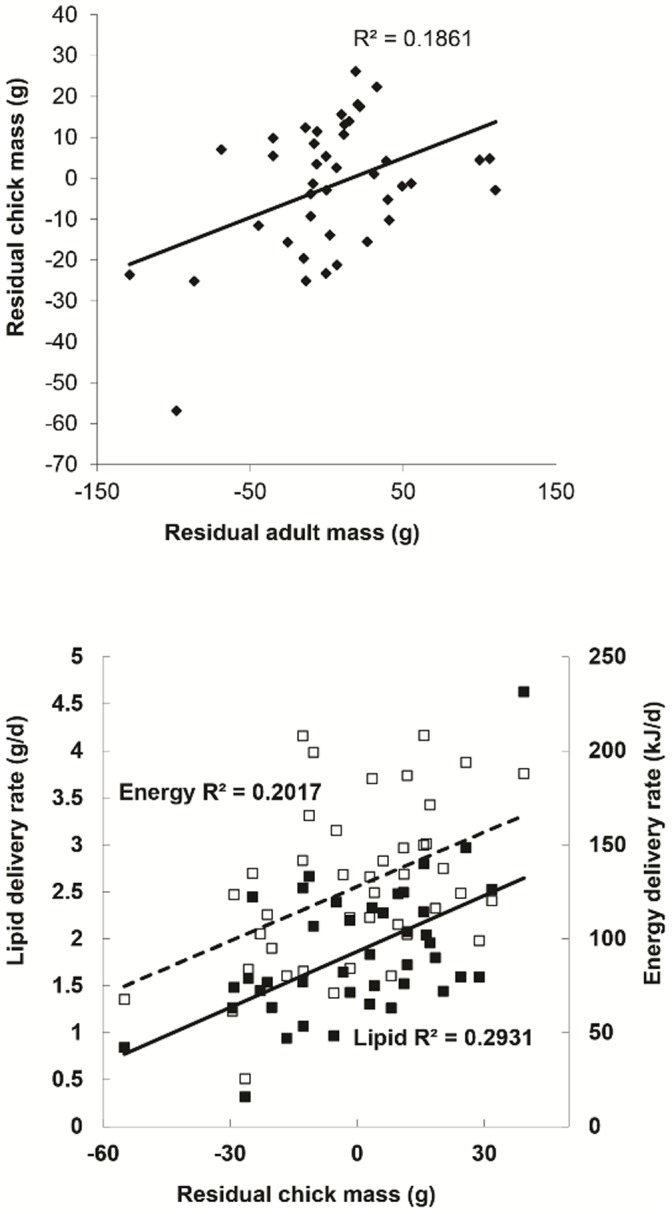
Chick mass gains are affected by food delivery rates and parental body mass. **a.** Residual of chick mass on chick age increases with residual of adult mass on chick age. **b.** Lipid (filled symbols, filled line) and energy (unfilled symbols, dashed line) delivery rates increase with the residual of chick mass on chick age. Values shown are the residuals relative to the residuals for the same individual on the first day of the experiment.

Compared with controls, dive depth and duration were smaller, while surface pause for a given dive depth was longer for single-handicapped birds (Table 3). Handicapped birds spent less time under water per day (Table 3). Chick growth rates increased with lipid delivery rates, energy delivery rates and adult body mass (Fig. 2). Handicapped birds tended to deliver more less-risky and fewer more-risky prey items (Table 5). Handicapped birds also tended to bring fewer shallow benthic prey items (Table 5). Partners tended to deliver fewer less-risky prey items than their mates (Table 5).

**Table 5:** Prey delivered by handicapped parental thick-billed murres or partners of those handicapped individuals at Coats Island in 2004.

	Less-risky (small capelin)	Less-risky (invertebrates)	More-risky (schooling)	Shallow benthics	Other
Male	7.9±1.6%*	−1.9±4.6%	−9.4±2.2%*	−8.3±2.5%*	11.7±5.7%
Female	11.4±4.2%*	15.9±5.3%*	−8.1±2.6%*	−9.9±1.5%*	−9.3±6.9%
Male partners	−12.4±3.6%*	1.4±4.8%	0.4±1.8%	−2.2±1.9%	12.8±7.1%
Female partners	−8.1±2.9%*	1.2±4.1%	0.2±2.2%	0.7±2.1%	6.0±4.3%

Values are differences in percentage delivered relative to control murres. Values in bold and marked with an asterix depart significantly from controls (ANOVA with post-hoc *t*-test).

Chick growth rates differed among treatments, and the effect of each treatment depended on the sex of the handicapped parent (Table 3, Fig. 1a). Chick growth rates were lower for those of doubly-handicapped and singly-handicapped male parents than for the offspring of control pairs, but the chicks of handicapped females (both single and double-handicaps) did not differ from controls. Likewise, energy delivery rates and lipid delivery rates differed among treatments, and were lower than controls for doubly-handicapped birds but not for singly-handicapped birds (Table 3, Fig. 1b). The differences in chick wing length between treatments were not significant (2003: F_1,35_ = 0.54, P = 0.3; 2004: F_1.37_ = 2.2, P = 0.06). Partners did not compensate for reduced feeding rates as there were no differences between the partners of handicapped birds and control birds in energy or lipid delivery rates (Table 3, Fig. 1b). There was also no relationship between the energy or lipid delivery rate of handicapped birds and those of their partners (energy delivery rate: double handicap: R^2^ = 0.03, t_14_ = 0.55, P = 0.59; single handicap: R^2^ = 0.05, t_14_ = −0.79, P = 0.44; combined: R^2^ = 0.00, t_30_ = −0.18, P = 0.86; lipid delivery rate: double handicap: R^2^ = 0.03, t_14_ = 0.60, P = 0.63; single handicap: R^2^ = 0.04, t_14_ = −0.71, P = 0.47; combined: R^2^ = 0.01, t_30_ = −0.21, P = 0.84).

### Sex Differences

While there were few differences between males and females in most parameters (Table 3), chicks gained less mass when their father was handicapped than when their mother was handicapped (single handicap–male: −2.59±4.24 g, female: 10.71±3.71 g, t_29_ = 2.69, P = 0.01; control–male: 13.6±3.6 g, female: 9.45±4.50 g, t_28_ = 1.07, P = 0.29; see also Table 3 and Fig. 1). Likewise, handicapped males, but not females, showed higher lipid mobilisation (Table 3). Handicapped females delivered more less-risky prey than controls (Table 4) and both sexes delivered less more-risky prey items and shallow benthic fish.

## Discussion

The effects of handicapping adult thick-billed murres reverberated within families. In response to reduced foraging efficiency, parents displayed reduced energy (lipids) availability to both themselves and their chicks, as measured by body mass (both sexes) and lipid mobilisation (in males). Behavioural changes by the parents led to a change in the distribution of lipid stores within the family, as detected by reduced adult mass, lower lipid delivery rates and altered neutral lipid flux for males. As is the case with most long-lived species, the cost of increased parental investment was partly passed on to the chicks [64], [7], [10], [11], [12]. Chicks of handicapped parents grew slower because handicapped parents fed their chicks less (Figs. 1, 2), as is the case in penguins ([65], but see [66]). Handicapping had a smaller effect on chick wing growth, which is largely maintained during very poor years at the expense of body growth, as wing growth is essential for fledging [67], [68]. Murre chicks must cope with an abrupt transition to life at sea three weeks after hatching, and the accumulation of energy stores is likely to be necessary for survival during the post-departure period, especially within the colony halo where food abundance is low [58]. Larger chicks have higher post-fledging survival in many birds [69], [70], [71], [72], [38].

Handicapped adults had lower energy stores (lower body mass, higher lipid mobilisation in males) than control adults, reflecting increased investment (buffering) to compensate for reduced foraging performance. Nonetheless, the magnitude of handicap seemed to play a role because wing-clipping had less effect than single floaters (mass loss, chick growth rates in males), and single floaters often had less of an effect (or no significant effect) than two floaters (chick growth rates in males, energy delivery rates, mass loss, Fig. 1). The size of lipid stores also affects behaviour in other birds, including rate of abandonment and chick-provisioning rates [73], [74], [75], [15]. Thus, to some degree, adults reduced their own body mass to increase the growth of their chicks by transferring their own lipid stores to their chicks via higher time spent foraging. Offspring of adults with higher body mass grew quicker (Fig. 2), likely because individual quality is an important part of lifetime reproductive success in long-lived birds [76], [77]. Furthermore, resighting probabilities were reduced, at least for doubly-handicapped individuals. Differences in resighting frequency might have occurred because of emigration from the area of observation, or because of a reduction in post-breeding survival, and shows that reproductive investment may affect subsequent likelihood of return [10], [78].

Similar to other studies of auks, investment strategies may have differed between the sexes as chick growth was slower when males were handicapped than when females were [10], [11], [12]. Unhandicapped female murres provision more and lose more mass than unhandicapped males, presumably because males maintain body condition for the post-fledging period of male-only care (reviewed in [38]). Given that the male-only increase in neutral lipids suggests an increase in lipid mobilisation by males, we propose that males facing an environmental challenge maintain energy stores to increase their ability to rear the chick post-fledging. Our results therefore challenge our initial prediction that males have a fixed investment strategy. Alternatively, the handicaps may have had a greater impact on male foraging strategies than female foraging strategies, as males forage at shallow depths at night, and therefore may be more impacted by alterations in buoyancy [38]. Handicapped individuals of both sexes increased capture of less-risky prey items and decreased capture of more-risky prey items, suggesting that the response to an increase in energy costs is to switch to feeding on less-risky prey. The prey delivered most often by males, such as shallow benthic fish, other small fish and invertebrates, are captured at shallow depths or during extended activity underwater [61], [38] and buoyancy- or drag-increasing handicaps may have had a proportionally greater impact under those conditions (see strong effects on those prey groups in Table 4).

In contrast to other studies of auks [79], [10], [11], [12], we found no compensation by partners of handicapped birds. Among five recent studies in seabirds, three have shown a compensatory response from the partners of handicapped birds [10], [11], [80] and two did not find such a response ([66] and our own). Consequently, it seems that compensatory behaviour is not consistent and may depend on the overall availability of food in a given year.

Corticosterone also did not increase for handicapped parents, at least for single handicaps despite their higher mass loss, higher neutral lipids for males and lower feeding rates. Corticosterone increased during handicapping in another auk [11], and in murres handicapped for an entire year [81], but not in penguins [82], [83], or in three studies where birds were equipped with loggers for short periods [84], [85], [86], [81]. Corticosterone decreased with date at our study site (see Table 3), despite evidence that feeding conditions deteriorated with date and chick demands increased [58]. Corticosterone levels are often higher in anticipation of foraging activity [87], [88], [89], and time spent foraging (flying/diving) doubles around the time of hatch [90], [37], [91]. Perhaps extenuating factors obscured small effect sizes associated with corticosterone and partner feeding rates; positive effects might have been observed with larger samples. Alternatively, corticosterone may be modulated according to the glucose needs of the parent, which may not have been directly affected by our handicaps [82]. Moreover, corticosterone dynamics may be associated with dramatic mass loss occurring at the time of hatch [92], [37].

Large scale climatic changes or local disturbances may cause changes in prey availability [93],[3]. Because they integrate information over large oceanic regions, seabirds are useful indicators of marine changes [94], [95], [96], [9]. Experimental studies, such as our own and those of Gill et al. (2002), are useful for pinpointing what metrics are most useful and for understanding the mechanisms underlying correlations between environmental proxies and their impacts on seabirds [11], [12], [66], [83]. For example, due to earlier ice break-up in Hudson Bay, murres at our study site have switched from larger to smaller prey items and chicks grow less quickly [43], [44], [45]; energy costs for delivery of many small items is greater than a single large item [46]. The lower return rate of handicapped adults in our study suggests that ice retreat may lead to reduced adult survival through increased effort to provision offspring. To date, studies of murres have only detected an effect of environmental change on adults when those effects are particularly dramatic [97], [98], [99]. Our results support the idea that long-lived seabirds, chick growth rates and adult foraging behaviour are likely to be more sensitive indicators of changes in prey abundance ([94], [100], [95], [97], our study where chick growth and adult diving were the only parameters showing similar trends across treatments). Some adult metrics, such as adult mass or plasma neutral lipids may also serve as useful early warning signs [101].
